# Trends in the Cumulative Incidence of Vocational Rehabilitation Indicators in Brazil, 2007–2016

**DOI:** 10.3390/ijerph17113952

**Published:** 2020-06-03

**Authors:** Cristiano Barreto de Miranda, João Silvestre Silva-Junior, Gisele Aparecida Fernandes, Frida Marina Fischer

**Affiliations:** 1 Graduate Public Health Program, School of Public Health, University of São Paulo, São Paulo 01246-904, Brazil; 2 Department of Medicine, São Camilo University Center, Av. Nazaré, 1501, Ipiranga, São Paulo 04263-200, Brazil; joao.junior@prof.saocamilo-sp.br; 3 Group of Epidemiology and Statistics on Cancer, AC Camargo Cancer Center, Rua Tagua, 440, São Paulo 01508-010, Brazil; giseleunifei@gmail.com; 4 Department of Environmental Health, School of Public Health, University of São Paulo, São Paulo 01246-904, Brazil; fischer.frida@gmail.com

**Keywords:** vocational rehabilitation, disability, social security, occupational health

## Abstract

Vocational rehabilitation (VR) aims at improving work ability to facilitate workers’ return to work. VR is provided in Brazil by the public social security system. The aim of the present study was to analyze trends in VR indicators for Brazil from 2007 to 2016. Based on open-access, secondary aggregate data, we calculated the cumulative incidence of VR indicators. We fitted Prais-Winsten generalized linear regression models to estimate trends and calculated annual percent variation with the corresponding 95% confidence interval (95% CI). The mean cumulative incidence of referrals to VR services was 37.16/1000 temporary disability benefits granted and exhibited a decreasing trend of −6.92% (95% CI: −8.38; −5.43). The mean cumulative incidence of admissions to VR services was 57.34/100 referrals and exhibited an increasing trend of 3.31% (95% CI: 1.13; 5.53). The mean cumulative incidence of rehabilitation was 57.43/100 admissions and remained stable along the analyzed period, −2.84 (95% CI: −5.87; 0.29). Our findings evidence a reduction in the number of workers referred for VR, an increase of admissions, and stability in the cumulative incidence of rehabilitated workers.

## 1. Introduction

Vocational rehabilitation (VR) refers to a multi-professional approach that is provided to individuals of working age with health-related impairments, limitations or restrictions to optimize work participation [1]. Available evidence points to the efficacy of VR to maximize work ability, reduce the number of missed workdays and early retirement, accelerate return to work, and contain social security costs [2,3,4,5]. While the approach to VR varies substantially among countries, the odds of successfully returning to and remaining at work are higher for programs which include multi-disciplinary actions [6,7].

The World Health Organization launched in 2017 “Rehabilitation 2030: A Call for Action”, which calls for coordinated action among Member States, international and professional organizations, non-governmental organizations and rehabilitation experts to strengthen rehabilitation services [8]. Two years later, WHO held the “Second Global Rehabilitation 2030 Meeting”, in which attention was called to the global need for rehabilitation services and research, including VR, particularly in low- and medium-income countries as a function of an increasing prevalence of noncommunicable diseases and limited resources for rehabilitation [9]. Work disability results in a high socioeconomic burden not only for workers, but also for employers and society at large [10]. For this reason, increasing the participation of individuals with disabilities in the labor force should have high priority in the global scientific and political agenda.

In Brazil, a governmental VR program is provided by the National Institute of Social Security (Instituto Nacional do Seguro Social—INSS), namely, an autarchic federal agency within the public social security system. VR includes educational, adjustment and re-adjustment support to facilitate return to work, especially among workers who receive work disability benefits due to disease or accidents [11,12]. This is a decentered program implemented by social security offices distributed all across the country. Until 2018, staff was comprised of medical legal experts, social security analysts—known as reference agents, who include social workers, psychologists, physical and occupational therapists—and social security technicians charged with managerial services. The essential tasks of teams included: assessing the work potential of individuals referred for VR, channeling, following up and seeking community support for VR resources, defining the conclusion of VR processes, and surveying long-term outcomes in terms of employment maintenance [13]. 

Since monitoring and analyzing information on VR services might be relevant to assess the INSS program and identify eventual hindrances, the aim of the present study is to analyze temporal trends of VR indicators for Brazil from 2007 to 2016. 

## 2. Materials and Methods 

This was an ecological study of time series relative to national and macro-regional indicators of INSS VR services. We analyzed data available at a public social security database (http://www3.dataprev.gov.br/infologo/) corresponding to the period from 2007 to 2016. 

Cumulative incidence was calculated based on the nationwide and macro-regional annual numbers of: (a) workers who contributed to INSS, (b) temporary work disability benefits granted (sickness and accident pay), (c) individuals referred for VR, (d) individuals admitted to VR services, and (e) individuals who concluded VR. 

Annual national and macro-regional cumulative incidence rates were calculated for the following indicators:

- Benefit incidence (BI): number of benefits granted, divided by the number of workers who contributed to the social security system each year
- VR referral incidence (RI): number of workers first referred for VR, divided by the number of benefits granted each year
- VR admission incidence (AI): number of workers first admitted for VR, divided by the number of workers referred for VR each year
- Rehabilitated worker incidence (RWI): number of workers who concluded VR, divided by the number of workers admitted for VR each year.

To make the presentation of the results clearer, BI and RI were multiplied times 1000 and AI and RWI times 100.

The cumulative incidence of each indicator was calculated according to methodological recommendations by Antunes and Waldman [14]. We estimated the annual percent change (APC) by means of Prais-Winsten generalized linear regression, which allows correcting first order autocorrelation in time-series analysis. As a result, we were able to rate trends as increasing (*p* ≤ 0.05) and positive regression coefficient), decreasing (*p* ≤ 0.05 and negative regression coefficient) or stable (*p* > 0.05) as well as to measure annual percent increase or decrease of cumulative incidence rates with the corresponding 95% confidence interval, with a significance level of *p* ≤ 0.05. Trend analysis was performed for the entire country and each of its five macro-regions using software STATA 14.1 (College Station, TX, USA, 2015).

The present study is based on open-access aggregate data in the public domain as per the Brazilian law no. 12,527/2011 [15] and complies with the Brazilian National Health Council Resolution no. 466/2012 [16].

## 3. Results

A total of 14,928,579 temporary disability benefits were granted in Brazil along the analyzed 10 years (2007–2016). A total of 543,739 individuals were referred to INSS VR services, of whom 308,797 were admitted (56.8% of those referred) and 176,752 completed the program (57.2% of those admitted). 

The mean cumulative incidence of temporary disability benefits granted nationwide along the analyzed period was 23.90/1000 workers who contributed to INSS. The highest rate corresponded to the South region and the lowest to the Central-West. Only the Southeast region exhibited a decreasing trend of −3.33% (95% CI: −6.42; −0.14) in the rate of new disability benefits; this rate remained stable in all the other regions and also nationwide (Table 1).

The mean cumulative incidence of workers referred for VR was 37.16/1000 individuals granted temporary disability benefits. The rates corresponding to the Southeast and Central-West regions were higher than the national average. For the country as a whole, we detected a decreasing trend of −6.92% (95% CI: −8.38; −5.43) as well as for all macro-regions, being the North that had the highest negative annual percent variation (Table 1). 

The mean cumulative incidence of new admissions to VR was 57.34/100 referrals. Only the rate for the Southeast region was higher than the national average and exhibited an increasing trend, as was also the case of the Central-West. Relative to the country as a whole, we found an annual increasing trend of 3.31% (95% CI: 1.13; 5.53) in the cumulative incidence of admissions. In turn, the rates for the North, Northeast and South regions remained stable (Table 2). 

The cumulative incidence of rehabilitated workers was 57.43/1000 admissions. The rates for the North, Northeast and Central-West regions were higher than the national average, but the Central-West exhibited a decreasing trend of −7.29% (95% CI: −10.22; −4.27). The rates for the country as a whole and all other four macro-regions remained stable (Table 2).

## 4. Discussion

In the present study we found a decreasing trend in the number of workers referred for VR in Brazil from 2007 to 2016 even though there was no parallel significant decreasing trend in the number of individuals granted disability benefits. In turn, the rate of workers admitted for VR exhibited a nationwide increasing trend. Yet the rate of actual rehabilitated workers across the country remained stable along the analyzed period.

On a separate macro-region analysis, all five exhibited similar behavior in regard to new referrals, to wit, a decreasing trend. The number of admissions exhibited an increasing trend in the Southeast and Central-West, while it remained stable in the North, Northeast and South regions. In regard to completed rehabilitation, the Central-West exhibited a decreasing trend and remained stable in the other four regions. 

Our findings indicate that less than 4% of all workers granted temporary disability along 10 years were referred for a VR eligibility assessment. In Israel, for instance, workers with disabilities may request direct access to VR services [17]. Differently, in Brazil, referrals depend on the judgment of medical legal experts at the time of the examinations required to obtain temporary disability benefits; workers may also be directly referred upon a judge’s decision [13]. Therefore, the fact that the number of new benefits remained stable while that of referrals decreased might be due to a reduction in the number of workers medical legal experts rated as needing VR.

This decrease in the rate of referrals might also be accounted for by an increase in the number of workers who received disability retirement benefits. Indeed, this rate grew 9% nationwide from 2012 to 2016 by comparison to 2007–2011 [18,19,20,21]. This type of benefit is only granted to workers with permanent inability to perform any work-related activity, i.e., when there is not any possibility of VR. In other countries, like Finland [22], retiring workers are seldom referred to VR services. 

Referred workers are initially received at VR services by the managerial staff and scheduled for socio-professional assessment, which is performed by a designated reference agent. Decision making on admission is jointly made by the medical legal and socio-professional assessment teams [13]. Changes in the admission criteria may help explain the increasing trend in the nationwide rate of admissions. Alternatively, in November 2011, INSS published the first edition of “Technical Manual of Procedures in Vocational Rehabilitation”, which standardized and included new actions in INSS VR procedures [23]. Similarly, the number of workers who enrolled in VR programs at two services in Germany effectively increased in the period from 2011 to 2015 [24]. In Israel, 70% of workers with intellectual disabilities who requested VR were admitted [17]. 

Despite a stable nationwide cumulative incidence, the number of actual rehabilitated workers was low by comparison to the United States of America (USA). According to data reported by the Rehabilitation Services Administration of the United States Department of Education, their VR program resulted in annual increases in the number of individuals who achieved successful employment outcomes from 2011 to 2016 [25]. While this type of comparison is hindered by differences in social security systems and labor laws, analysis of the average number of participants in VR programs per year evidenced that the rate of successful outcomes was 10 times higher in the USA than in Brazil. In the former, 87% of 540,877 candidates were considered eligible in 2016 and 186,713 obtained employment [25]. Reasons for these more favorable outcomes include an annual investment in VR of more than USD 2.5 billion, promotion of scientific research and evidence-based practices [26]. A study that analyzed data from the National Vocational Rehabilitation Services Documentary System in Taiwan found that 68.7% of workers with disabilities were rehabilitated between 2008 and 2010 with successful employment outcomes [27]. 

Late onset of intervention might be one of the explanations for the low rates we found. Perhaps there is a long history of absenteeism and temporary disability benefits before workers are referred for VR. A Brazilian study showed the average time to admission into VR programs was 2.5 years [28]. In the Netherlands, for instance, interventions to reintegrate workers with disabilities into the labor force start much earlier, to wit, within the first six weeks, and follow a fixed process that is mandatory by law [29]. Scientific evidences showed, the longer the time out of work, the lower the odds to return [7]. Therefore, long intervals from the time of leave to admission to VR might hinder the success of the process.

Our findings raise concerns in regard to the access to VR services in Brazil and to the goal of increasing the odds of individuals with variable degrees of disability to find employment. Good work disability management practices consider social inclusion and health equity—particularly in the workplace [30]. In Brazil, actions by VR services are historically tied to macrostructural social security issues within an economistic and bureaucratic frame [31]. Public policies and actions are in contradiction, since the latter are often designed in a way to control social security costs at the expense of the constitutional rights of INSS-affiliated workers. 

In theory, to ensure efficacy to VR programs, INSS establishes partnerships and technical cooperation agreements with public and private organizations to provide services such as therapeutic assistance, initiatives to improve the educational level of workers, training and professional education. INSS is charged with providing the material resources needed, such as prostheses and orthoses, paying course, professional and working fees, transport and meal vouchers, and day rates [13]. As a result, the links each individual VR service builds to other social actors may have positive or negative influences on the implementation and effectiveness of the program. These factors may have possibly contributed to decreasing the incidence of successful outcomes in the Central-West region. Lack of integrated public policies, particularly within the Brazilian public health system, hinder the possibilities of achieving better VR outcomes [32].

INSS issues a certificate to all individuals who complete VR programs that describes the dates of onset and conclusion, courses attended/training received, and jobs for which they were specifically rehabilitated [13]. These certificates enable rehabilitated workers to be candidates for disability quotas as established by the Brazilian legislation [11]; a similar procedure was implemented in Taiwan [27]. However, certificates do not ensure sustainable return to work. Analyzing this outcome requires longitudinal studies after the end of VR programs.

There are differences between countries in the rates of re-entry into the labor market of people with disabilities, according to compensation policies [33]. A Brazilian study performed with 802 workers found that only 29.4% of those who had undergone VR were employed one year after the end of the program [28], which may be seen as a result of shortcomings in INSS accredited services. In the United States, the rate of individuals with considerable disabilities who achieved successful employment outcomes ranged from 93.5% to 94.1% from 2009 to 2016 [25]. In Sweden, 32.1% of workers with disabilities enrolled in VR services were still working two years after completing VR programs [34]. Differences among sustainable return-to-work program outcomes can be explained by workplace interventions and policies implemented in work incapacity programs, according to sociopolitical and institutional characteristics of implementing organizations, and with the collaboration of other stakeholders [30].

Although most individuals affiliated to INSS are private sector workers, the discussions on VR within the public sector seem to be rather similar from the nationwide perspective.

Our results should be analyzed bearing in mind the limitations intrinsic to the database searched, which only compiles secondary aggregate data and does not provide detailed information. As a result, there are shortcomings in sociodemographic, occupational and clinical information. Yet, this is the single source of open access official data on INSS VR services. A second limitation—impossibility of drawing individual causal inferences—is inherent to ecological studies. Nevertheless, relevant population-based inferences are indeed possible, and our results point to the need to improve access and VR for disabled workers in Brazil.

## 5. Conclusions

The results of the present study evidence a decreasing trend in the referral of disabled workers to VR services in Brazil, despite a large number of granted disability benefits. We further detected an increasing trend of admissions into services among referred workers and stability in the rate of those who conclude the program. Analysis per macro-region facilitated our understanding of the progression of the analyzed indicators according to regional aspects. 

A reduced rate of referrals means that less workers have access to services likely to help them return to work. In turn, high admission rates together with stability in the number of workers who conclude programs suggest there is a wait list for a place in VR services. These findings point to a lack of effectiveness of VR as a strategy to reduce the time out of work through early return to work in Brazil.

Our study contributes with new data to the analysis of the national and regional performance, along a decade, of social services charged with assisting people with variable degrees of disability for work. Analysis points to the need for more thorough studies of VR indicators at the individual and organizational level in Brazil to improve these services, since they are essential to the national social security system. 

## Figures and Tables

**Table 1:** Absolute and relative frequencies and mean cumulative incidence rates and trends relative to temporary disability benefits granted and workers referred to National Institute of Social Security (INSS) vocational rehabilitation services. Brazil and macro-regions, 2007–2016.

Place	*N*	%	Mean Cumulative Incidence (/1000)	APC ^a^ (95% CI) ^b^
Granted temporary disability benefits				
North	747,585	5.01	24.98	2.58 (−1.56; 6.90)
Northeast	2,865,269	19.19	27.61	2.74 (−0.93; 6.54)
Southeast	6,741,142	45.16	20.47	−3.33 (−6.42; −0.14) *
South	3,645,869	24.42	32.89	0.10 (−0.52; 0.72)
Central-West	928,714	6.22	18.22	0.92 (−1.66; 3.58)
Brazil	14,928,579		23.90	−0.74 (−3.40; 2.00)
Workers referred for VR				
North	24,826	4.57	35.78	−9.97 (−17.26; −2.05) *
Northeast	97,304	17.89	35.86	−8.50 (−10.27; −6.70) *
Southeast	266,702	49.05	39.68	−7.14 (−9.03; −5.22) *
South	118,764	21.84	32.95	−4.31 (−8.13; −0.33) *
Central-West	36,143	6.65	39.66	−4.17 (−6.16; −2.13) *
Brazil	543,739		37.16	−6.92 (−8.38; −5.43) *

Caption: ^a^ annual percent change; ^b^ 95% confidence; * *p* < 0.05.

**Table 2:** Absolute and relative frequencies and mean cumulative incidence rates and trends relative to workers admitted to and rehabilitated at INSS vocational rehabilitation services. Brazil and macro-regions, 2007–2016.

Place	*N*	%	Mean Cumulative Incidence (/100)	APC ^a^ (95% CI) ^b^
Admissions				
North	11,048	3.58	47.24	10.88 (−2.23; 25.74)
Northeast	53,457	17.31	55.09	5.96 (−0.35; 12.68)
Southeast	161,794	52.39	66.68	3.52 (1.87; 5.19) *
South	65,090	21.08	54.27	−1.14 (−3.93; 1.72)
Central-West	17,408	5.64	48.73	3.96 (1.13; 6.86) *
Brazil	308,797		57.34	3.31 (1.13; 5.53) *
Rehabilitated workers				
North	7,471	4.23	77.70	−5.54 (−15.27; 5.31)
Northeast	34,077	19.28	66.48	−3.92 (−8.07; 0.41)
Southeast	92,532	52.35	56.74	−2.88 (−5.82; 0.14)
South	32,067	18.14	51.69	−0.34 (−5.23; 4.81)
Central-West	10,605	6.00	63.67	−7.29 (−10.22; −4.27) *
Brazil	176,752		57.43	−2.84 (−5.87; 0.29)

Caption: ^a^ annual percent change; ^b^ 95% confidence; * *p* < 0.05.

## References

[1] Escorpizo R., Reneman M.F., Ekholm J., Fritz J., Krupa T., Marnetoft S.-U., Maroun C.E., Guzman J.R., Suzuki Y., Stucki G. (2011). A Conceptual Definition of Vocational Rehabilitation Based on the ICF: Building a Shared Global Model. J. Occup. Rehabil..

[2] Disler P.B., Pallant J.F. (2001). Vocational rehabilitation. BMJ.

[3] Hoefsmit N., Houkes I., Nijhuis F.J.N. (2012). Intervention Characteristics that Facilitate Return to Work After Sickness Absence: A Systematic Literature Review. J. Occup. Rehabil..

[4] Allaire S.H., Li W., LaValley M. (2003). Reduction of job loss in persons with rheumatic diseases receiving vocational rehabilitation: A randomized controlled trial. Arthritis Rheum..

[5] Petit A., Fouquet N., Roquelaure Y. (2015). Chronic low back pain, chronic disability at work, chronic management issues. Scand. J. Work. Environ. Health.

[6] Berglund E., Anderzén I., Andersén Å, Carlsson L., Gustavsson C., Wallman T., Lytsy P. (2018). Multidisciplinary Intervention and Acceptance and Commitment Therapy for Return-to-Work and Increased Employability among Patients with Mental Illness and/or Chronic Pain: A Randomized Controlled Trial. Int. J. Environ. Res. Public Health.

[7] Marois E., Durand M.-J. (2009). Does participation in interdisciplinary work rehabilitation programme influence return to work obstacles and predictive factors?. Disabil. Rehabil..

[8] World Health Organization (2017). Rehabilitation 2030—A Call for Action.

[9] Feuerstein M., Findley P.A., Gross D.P. (2019). Reducing the Global Burden of Work Disability: A Call to Action to Support the World Health Organization’s Rehabilitation 2030. J. Occup. Rehabil..

[10] Loisel P., Durand M.-J., Diallo B., Vachon B., Charpentier N., Labelle J. (2003). From Evidence to Community Practice in Work Rehabilitation: The Quebec Experience. Clin. J. Pain.

[11] Brasil Lei nº 8.213, de 24 de julho de 1991. Dispõe sobre os Planos de Benefícios da Previdência Social e dá outras providências. Diário Oficial da União: Brasil, 1991. http://www.planalto.gov.br/ccivil_03/leis/l8213cons.htm.

[12] Brasil Decreto nº 3.048, de 06 de maio de 1999. Aprova o Regulamento da Previdência Social, e dá outras providências. Diário Oficial da União: Brasil, 1999. http://www.planalto.gov.br/ccivil_03/decreto/d3048.htm.

[13] Brasil (2018). Manual Técnico de Procedimentos da Área de Reabilitação Profissional.

[14] Antunes J.L.F., Waldman E.A. (2002). Trends and spatial distribution of deaths of children aged 12–60 months in São Paulo, Brazil, 1980–1998. Bull. World Health Organ..

[15] Brasil (2011). Lei nº 12.527, de 18 de novembro de 2011. Dispõe sobre os procedimentos a serem observados pela União, Estados, Distrito Federal e Municípios, com o fim de garantir o acesso a informações.

[16] Brasil Ministério da Saúde. Conselho Nacional de Saúde. Resolução nº 466, de 12 de dezembro de 2012. Dispõe sobre as diretrizes e normas regulamentadoras de pesquisas envolvendo seres humanos. Ministério da Saúde: Brasília, 2012. http://conselho.saude.gov.br/ultimas_noticias/2013/06_jun_14_publicada_resolucao.html.

[17] Hornik-Lurie T., Zilber N., Lerner Y. (2012). Trends in the use of rehabilitation services in the community by people with mental disabilities in Israel; the factors involved. Isr. J. Health Policy Res..

[18] Brasil (2009). Ministério da Previdência Social. Anuário Estatístico da Previdência Social.

[19] Brasil (2012). Ministério da Previdência Social. Anuário Estatístico da Previdência Social.

[20] Brasil (2015). Ministério da Fazenda. Anuário Estatístico da Previdência Social.

[21] Brasil (2017). Ministério da Fazenda. Anuário Estatístico da Previdência Social.

[22] Laaksonen M., Gould R. (2014). Return to Work After Temporary Disability Pension in Finland. J. Occup. Rehabil..

[23] Brasil (2011). Manual técnico de procedimentos da área de reabilitação profissional.

[24] Nivorozhkin A., Reims N., Zollmann P., Bethge M. (2018). Leistungen zur Teilhabe am Arbeitsleben—Rehabilitanden der Bundesagentur für Arbeit und der Deutschen Rentenversicherung im Vergleich. Die Rehabil..

[25] U.S. Department of Education (2019). Rehabilitation Services Administration Report for Fiscal Year 2016.

[26] Dutta A., Gervey R., Chan F., Chou C.-C., Ditchman N. (2008). Vocational Rehabilitation Services and Employment Outcomes for People with Disabilities: A United States Study. J. Occup. Rehabil..

[27] Jang Y., Wang Y.-T., Lin M.-H. (2013). Factors Affecting Employment Outcomes for People with Disabilities Who Received Disability Employment Services in Taiwan. J. Occup. Rehabil..

[28] Vacaro J.E., Pedroso F.S. (2011). Performance of insured workers in the rehabilitation service at the National Institute for Social Security. Acta Fisiátr..

[29] Mittag O., Kotkas T., Reese C., Kampling H., Groskreutz H., De Boer W., Welti F. (2018). Intervention policies and social security in case of reduced working capacity in the Netherlands, Finland and Germany: A comparative analysis. Int. J. Public Health.

[30] Ståhl C., Costa-Black K., Loisel P. (2017). Applying theories to better understand socio-political challenges in implementing evidence-based work disability prevention strategies. Disabil. Rehabil..

[31] Takahashi M.A.B.C., Iguti A.M. (2008). As mudanças nas práticas de reabilitação profissional da Previdência Social no Brasil: Modernização ou enfraquecimento da proteção social?. Cadernos de Saúde Pública.

[32] Maeno M., Vilela R.A.D.G. (2010). Reabilitação profissional no Brasil: Elementos para a construção de uma política pública. Rev. Brasileira de Saúde Ocup..

[33] Anema J.R., Schellart A.J.M., Cassidy J.D., Loisel P., Veerman T.J., Van Der Beek A.J. (2009). Can Cross Country Differences in Return-to-Work After Chronic Occupational Back Pain be Explained? An Exploratory Analysis on Disability Policies in a Six Country Cohort Study. J. Occup. Rehabil..

[34] Ahlgren Å., Bergroth A., Ekholm J., Schüldt K. (2007). Work resumption after vocational rehabilitation: A follow-up two years after completed rehabilitation. Work.

